# Dosimetric Predictors of Problematic Receptive Anal Intercourse After Prostate Radiation Therapy

**DOI:** 10.1016/j.adro.2026.102038

**Published:** 2026-04-03

**Authors:** Daniel R. Dickstein, Thodori Kapouranis, Andre Williams, Victoria Olsen, Kathryn E. Flynn, Robert Stewart, Barry S. Rosenstein, Tian Liu, Richard Stock, Rendi Sheu, Michael Lovelock, Andrew Jackson, Karyn A. Goodman, Deborah C. Marshall

**Affiliations:** a Department of Radiation Oncology, Icahn School of Medicine at Mount Sinai, New York, New York; b Department of Population Health Science and Policy, Icahn School of Medicine at Mount Sinai, New York, New York; c Division of Anatomy, Department of Surgery, University of Toronto, Toronto, Ontario, Canada; d Department of Medicine, Medical College of Wisconsin, Milwaukee, Wisconsin; e Department of Medical Physics, Memorial Sloan Kettering Cancer Center, New York, New York

## Abstract

**Purpose:**

While the effects of prostate radiation therapy on erectile function are well documented, data on receptive anal intercourse (RAI) remain sparse, despite its prevalence among gay and bisexual men. This study investigated associations between radiation dose to RAI functional anatomy and problematic RAI to identify preliminary, clinically meaningful dosimetric parameters.

**Methods and Materials:**

Fourteen prostate cancer survivors who engaged in RAI and completed Patient-Reported Outcomes Measurement Information System questionnaires were retrospectively evaluated. RAI functional anatomy (anal canal, levator ani, erectile tissues, neurovascular bundles, and internal pudendal arteries [IPAs]) was delineated, and dosimetric parameters were extracted. Dose-outcome relationships were assessed using Spearman's correlation (ρ) and logistic regression.

**Results:**

Anal canal maximum dose (Dmax; minimum dose to the hottest 0.03 cm3) and minimum dose tot he hottest 2 cm3 (D2cm3) were strongly associated with orgasm ability (ρ = −0.63), while mean dose (Dmean) showed a moderate association (ρ = −0.57); all anal canal parameters were moderately associated with orgasm pleasure (ρ range, −0.50 to −0.56). Levator ani Dmean and D2cm3 were moderately associated with orgasm ability and pleasure (ρ range, −0.48 to −0.58). Erectile tissue high-dose parameters were strongly associated with orgasm ability (ρ range, −0.61 to −0.68) but not with orgasm pleasure (ρ range, −0.39 to −0.46). IPA Dmax was moderately associated with orgasm ability (ρ = −0.52) and pleasure (ρ = −0.55). Neurovascular bundle dosimetry and anal pain showed no evidence of association. Exploratory dose thresholds corresponding to a 10% probability of dysfunction, in equivalent doses in 2 Gy fractions with an α/β ratio of 3 (EQD2[3]), were identified for the anal canal (Dmax, 40; Dmean, 9; D2cm3, 4), levator ani (Dmean, 31), erectile tissues (Dmax, 20; Dmean, 5), and IPA (Dmax, 35).

**Conclusions:**

This study provides the first evidence on the association between radiation dose to RAI functional anatomy and problematic RAI. While exploratory, these results may help guide future studies and inform counseling.

## Introduction

Up to 70% of prostate cancer survivors report treatment-related sexual dysfunction after radiation therapy.^1^ However, while the effects of radiation on penile erectile function are well documented,^2^, ^3^, ^4^ data on receptive anal intercourse (RAI) remain limited,^5^ despite its prevalence among gay and bisexual cisgender men.^6^

In the United States, approximately 31,000 gay and bisexual men are diagnosed with prostate cancer annually,^7^, ^8^, ^9^ and as many as 24,800 may engage in RAI,^6^ comparable to the estimated 25,000 annual brain and central nervous system tumor diagnoses.^8^ Yet, only 6% of gay and bisexual men report discussing RAI with their prostate cancer provider, let alone how treatment may affect it.^5^

During RAI, sexual pleasure is facilitated by the prostate, the sensate anus, the erectile tissues, the pelvic floor muscles, and the surrounding neurovasculature.^5^, ^6^, ^7^ After radiation, individuals may experience difficulties with RAI,^5^^,^^7^ termed problematic RAI,^6^ encompassing anodyspareunia (painful RAI), arousal dysfunction, orgasm dysfunction, and decreased sexual desire.^6^ Radiation-related problematic RAI may result not only from direct prostate injury but also from off-target radiation damage to surrounding structures that facilitate RAI.^2^, ^3^, ^4^^,^^10^, ^11^, ^12^

Extensive research has shown that off-target radiation can damage the functional anatomy required for penile erections, leading to the development of dose constraints and strategies to both mitigate and treat erectile dysfunction.^2^, ^3^, ^4^^,^^10^^,^^11^ In contrast, no studies have examined the effects of prostate radiation therapy on the functional anatomy involved in RAI, nor are there any evidence-based interventions to prevent or treat problematic RAI.^13^ Understanding the relationship between off-target radiation dose to RAI functional anatomy and problematic RAI would help establish clinically meaningful dose thresholds to mitigate problematic RAI and inform personalized treatment recommendations.

This study investigated the association between off-target radiation dose to RAI functional anatomy and problematic RAI, aiming to identify preliminary, clinically meaningful dosimetric parameters to minimize toxicity and improve quality of life.

## Methods and Materials

### Study design and participants

We conducted a cross-sectional, posttreatment analysis of the association between the radiation dose to RAI functional anatomy and problematic RAI in prostate cancer survivors with intact prostates who presented for follow-up at least 6 months after radiation treatment between June 2022 and August 2023. Patients treated with androgen deprivation therapy (ADT) were included if they had completed treatment with testosterone recovery. All patients reported engaging in RAI within the past 30 days. The study was approved by the institutional review board with a waiver of informed consent.

### Variables and outcome measurements

Patients self-reported sexual orientation,^14^ gender identity,^14^ sex recorded at birth,^14^ and sexual behaviors, including RAI.^15^ Problematic RAI was assessed using select items from the Patient-Reported Outcomes Measurement Information System (PROMIS) Sexual Function and Satisfaction version 2.0 questionnaire,^16^ which included orgasm ability, orgasm pleasure, and anal pain during RAI (Table E1). Baseline RAI function data were unavailable, and longitudinal changes in RAI were not assessed. The electronic medical record was reviewed for clinical variables, including gastrointestinal disorders (structural gastrointestinal diseases, disorders of gut-brain interaction, and inflammatory gastrointestinal diseases)^6^ present prior to radiation therapy and at the time of completion of the PROMIS Sexual Function and Satisfaction questionnaire.

### Anatomic delineation and dose extraction

For 14 patients, RAI functional anatomy, including the anal canal,^17^ levator ani,^18^ erectile tissues^10^ (proximal crura^2^ and penile bulb^2^), neurovascular bundles,^11^ and the internal pudendal arteries (IPAs),^3^ was delineated on treatment planning computed tomography (CT) fused with multiparametric magnetic resonance imaging (Table 1).

**Table 1:** Receptive anal intercourse anatomy contour definitions.

Anatomic structure*	Sexual function	Contour definition	Imaging used
Anal canal	Tightening around an inserted object; pleasurable sensations; contraction for orgasm	Cranial: anorectal junction, defined on sagittal T2-weighted MRI as the imaginary line connecting the inferior sacral and pubic bones Caudal: anal verge, defined by consensus from contour reviewers Note: the anal contour includes the internal and external anal sphincters	T2-weighted MRI IAS: iso/hypointense EAS: hypointense
Levator ani	Facilitates the ability to receive an inserted object; contraction for orgasm	Cranial: sacrospinous ligament Caudal: inferior pubic ramus, where levator ani fibers blend with the EAS Anterior: attachment to the pubic symphysis Posterior: coccyx Lateral: obturator internus muscle Medial: anal canal and urogenital hiatus Note: the levator ani includes the puborectalis, pubococcygeus, and iliococcygeus muscles	T2-weighted MRI Levator ani: hypointense
Erectile tissues	Arousal	*Corpus spongiosum (Penile bulb)* Cranial: corpora cavernosa divergence Caudal: urogenital diaphragm Anterior: urethra *Corpora cavernosa (Paired crura)* Cranial: divergence at the pubic arch is contoured as 2 separate structures Note: the corpus spongiosum and corpora cavernosa are contoured separately and subsequently booleaned together	MRI T2 FSE *Corpus spongiosum*: hyperintense *Corpora cavernosa*: hyperintense
Internal pudendal arteries	Blood supply and innervation to genitopelvic tissues	Cranial: sacrospinous ligament Caudal: crus, where it forms the common penile and scrotal arteries	MRI T2 FSE
Neurovascular bundles		Cranial: base of the seminal vesicles Caudal: urogenital diaphragm Anterior: prostate Posterior: rectum	MRI T2 FSE

*Abbreviations:* EAS = external anal sphincter; FSE = fast spin echo; IAS = internal anal sphincter; MRI = magnetic resonance imaging.

⁎ The prostate was excluded from the list of receptive anal intercourse anatomic structures because it was part of the radiation target.

For patients who received brachytherapy and external beam radiation therapy, high-dose-rate (HDR) planning CT or low-dose-rate (LDR) postimplant dosimetry CT were aligned with the external beam radiation therapy planning CT using rigid registration based on 4 landmarks: the anterior pubic symphysis, the posterior sacral edge, the superior portion of the pubic symphysis, and the anal verge.

Voxel doses were converted to the biologically effective dose (BED) using linear-quadratic radiobiological models. For external beam radiation therapy and HDR brachytherapy, BED was calculated using the linear-quadratic model for fractionated irradiation.^19^ For LDR brachytherapy, BED was calculated using the linear-quadratic model for continuous irradiation.^20^ For individuals treated with combined external beam radiation therapy and brachytherapy (HDR or LDR), the BED from each modality was summed to generate a composite BED.^21^

Composite and single modality BED were then converted to equivalent doses in 2 Gy fractions with an α/β ratio of 3 (EQD2[3]).^21^ We extracted the maximum dose (Dmax; minimum dose to the hottest 0.03 cm3) and mean dose (Dmean) for all structures, plus a minimum dose to the hottest 2 cm3 (D2cm3) for the anal canal and levator ani, and a minimum dose to the hottest 2% (D2%) for the IPAs, erectile tissues, and neurovascular bundles. Dosimetric parameters were selected a priori based on the radiation therapy dosimetry literature examining dose to functional genitopelvic anatomy and sexual health outcomes.^4^^,^^10^

### Statistical analysis

PROMIS raw scores were transformed to T-scores using the item-pattern response method. T-scores were compared with normative scores from the United States general population of sexually active adult men (range, 1-100; mean [SD], 50 [10]), with higher scores indicating greater concept intensity.^16^

Spearman’s correlation (ρ) assessed the relationship between dosimetric parameters and continuous T-scores. Strong and moderate ρ were defined as |ρ| > 0.6 and 0.59 > |ρ| > 0.4, respectively; ρ with *p* values < .1 were considered for further analysis.

For strong and moderate ρ between dosimetric parameters and continuous problematic RAI outcomes with *p* values < .1, logistic regression was used to evaluate associations with dichotomized problematic RAI outcomes. We dichotomized PROMIS outcomes using a clinically meaningful difference of at least 3 T-score points (approximately one-third of the SD) from the normative mean of 50. Dysfunctional orgasm ability and pleasure were defined as T-scores < 47, and dysfunctional anal pain as a T-score > 53.^22^

Dose thresholds (Gy EQD2[3]) were calculated for logistic regression models with *p* values < .1. A 10% predicted probability of dysfunction was selected as the illustrative threshold, as 10% has been judged a clinically relevant threshold for dysfunction in the absence of established tolerance doses in the prostate cancer radiation therapy toxicity literature.^23^

Because of the small sample size and hypothesis-generating aim, *p* values < .1 were considered exploratory evidence of association and were not interpreted as definitive statistical significance. All analyses were performed in R v4.3.1 (R Foundation for Statistical Computing).

## Results

Among 14 prostate cancer survivors with a median follow-up of 2.3 years (Q1-Q3, 1.3-3.5) after radiation therapy, 64% reported dysfunctional orgasm pleasure during RAI, 43% reported dysfunctional orgasm ability during RAI, and 29% reported anal pain during RAI. No patients had a documented gastrointestinal diagnosis. Radiation therapy modalities included stereotactic body radiation therapy (n = 3), intensity modulated radiation therapy (n = 2), brachytherapy combined with stereotactic body radiation therapy (n = 2) or intensity modulated radiation therapy (n = 6), and brachytherapy monotherapy (n = 1); 2 patients received a perirectal spacer (Table 2).

**Table 2:** Baseline clinical characteristics and sexual dysfunction rates in prostate cancer survivors who report receptive anal intercourse after radiation therapy.

Variable	All (N =14) n (%)
Age group (y)	
50-59	2 (14)
60-69	8 (57)
70-79	4 (29)
Sexual orientation	
Gay	13 (93)
Bisexual	1 (7)
Race	
White	10 (71)
Black	3 (21)
Asian	1 (7)
Ethnicity	
Hispanic	3 (21)
Non-Hispanic	11 (79)
Relationship status	
Single	11 (79)
Divorced/separated	1 (7)
Married/living with partner	2 (14)
Alcohol consumption	
Nondrinker	8 (57)
Drinks alcohol	6 (43)
Smoking status	
Never	7 (50)
Former	7 (50)
Current	0
Charlson comorbidity index	
0-2	0
3-4	8 (57)
≥5	6 (43)
PSA range (ng/mL)	
0-9.9	9 (64)
10-20	2 (14)
>20	3 (21)
Grade group	
1	3 (21)
2	3 (21)
3	4 (29)
4	2 (14)
5	2 (14)
Clinical stage	
T1	7 (50)
T2	6 (43)
T3 or T4	1 (7)
NCCN risk group	
Low	2 (14)
Intermediate	5 (36)
High	7 (50)
Radiation modality	
Pd-103 LDR BT	1 (7)
IMRT	2 (14)
SBRT	3 (21)
IMRT + Pd-103 LDR BT	5 (36)
SBRT + Pd-103 LDR BT	2 (14)
IMRT + Ir-192 HDR BT	1 (7)
BED continuous (Gy)	
Median (Q1-Q3)	198 (168–200)
EQD2 continuous (Gy)	
Median (Q1-Q3)	101 (77–108)
ADT	
Yes	6 (43)
ADT length (mo)	
Median (Q1–Q3)	11 (9–21)
Spacer	
Yes	2 (14)
Follow up, years	
Median (Q1-Q3)	2.3 (1.3–-3.5)
Orgasm ability	
Mean (95% CI)	46 (39, 54)
Dysfunction	6 (43)
Orgasm pleasure	
Mean (95% CI)	44 (37, 50)
Dysfunction	9 (64)
Anal pain	
Mean (95% CI)	57 (48, 67)
Dysfunction	4 (29)

*Abbreviations:* ADT = androgen deprivation therapy; BED = biologically effective dose; BT = brachytherapy; EQD2 = equivalent dose in 2 Gy fractions; HDR = high-dose-rate; IMRT = intensity modulated radiation therapy; Ir-192 = iridium-192; LDR = low-dose-rate; NCCN = National Comprehensive Cancer Network; Pd-103 = palladium-103; PSA = prostate-specific antigen; SBRT = stereotactic body radiation therapy.

**Figure 1:**
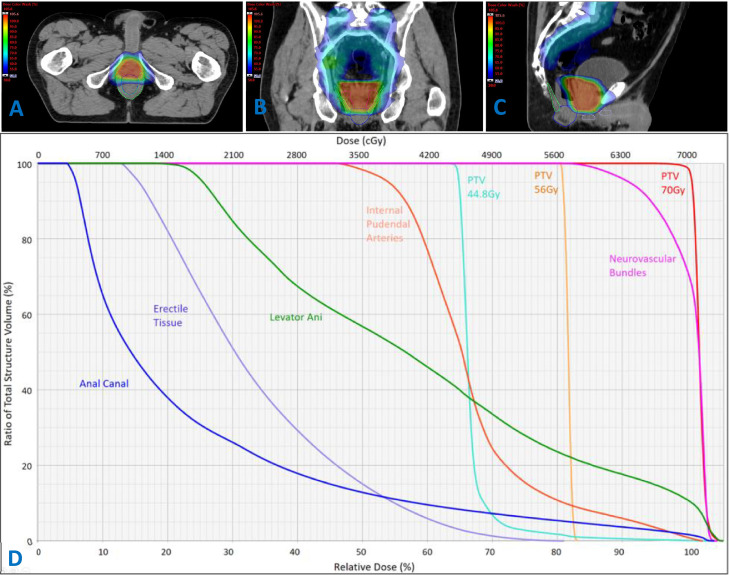
Off-target radiation dose to the receptive anal intercourse (RAI) functional anatomy. (A) Axial, (B) coronal, and (C) sagittal computed tomography images showing off-target radiation dose overlaid on RAI functional anatomy contours, including the anal canal (blue), erectile tissues (purple), levator ani (green), internal pudendal arteries (orange), and neurovascular bundles (pink). A 50% dose color wash is displayed to illustrate the spatial relationship between the prostate target and surrounding organs at risk. (D) Cumulative dose-volume histograms illustrating off-target radiation dose to RAI functional anatomy after prostate radiation therapy. *Abbreviation:* PTV = planning target volume.

Structures closer to the prostate received higher off-target radiation doses (Fig. 1 and Table 3). In exploratory dosimetric analyses, high-dose parameters were associated with problematic RAI across multiple anatomic structures. For the anal canal, Dmax and D2cm3 were strongly associated with orgasm ability (ρ, −0.63), and Dmean was moderately associated (ρ*,* −0.57). Anal canal dose parameters showed moderate associations with orgasm pleasure (ρ range, −0.50 to −0.56). Erectile tissue doses (Dmax, Dmean, and D2%) were strongly associated with orgasm ability (ρ range, −0.61 to −0.68) but not with orgasm pleasure (ρ range, −0.39 to −0.46). Levator ani Dmean and D2cm3 and IPA Dmax showed moderate associations with both orgasm ability and orgasm pleasure (levator ani ρ range, −0.48 to −0.58; IPA ρ range, −0.52 to −0.55). Neurovascular bundle dose was not associated with any outcomes, and anal pain was not associated with any dosimetric parameters (Table 4).

**Table 3:** Descriptive statistical distributions of dosimetric parameters for anatomic structures involved in receptive anal intercourse after prostate radiation therapy.

Dosimetric parameter	Mean (SD)	Median (Q1-Q3)
Anal canal		
Volume, cm3	36 (12)	32 (29-43)
Dmax (D0.03cm3), Gy	60 (18)	62 (53-74)
Dmean, Gy	6 (4)	5 (3-8)
D2cm3, Gy	21 (14)	16 (12-26)
Levator ani		
Volume, cm3	40 (9)	40 (34-44)
Dmax (D0.03cm3), Gy	234 (234)	149 (84-218)
Dmean, Gy	34 (9)	34 (29-39)
D2cm3, Gy	86 (25)	81 (79-94)
Erectile tissue		
Volume, cm3	10 (3)	9 (8-11)
Dmax (D0.03cm3), Gy	42 (29)	40 (15-58)
Dmean, Gy	12 (10)	9 (5-18)
D2%, Gy	37 (28)	30 (12-53)
Internal pudendal arteries		
Volume, cm3	8 (2)	8 (6-9)
Dmax (D0.03cm3), Gy	55 (23)	52 (42-69)
Dmean, Gy	28 (11)	32 (21-34)
D2%, Gy	49 (21)	48 (37-61)
Neurovascular bundles		
Volume, cm3	8 (2)	8 (6-9)
Dmax (D0.03cm3), Gy	207 (250)	103 (82-150)
Dmean, Gy	71 (14)	72 (60-81)
D2%, Gy	139 (112)	93 (81-129)

*Abbreviations:* D0.03cm3 = minimum dose to the hottest 0.03 cm3; D2% = minimum dose to the hottest 2%; D2cm3 = minimum dose to the hottest 2 cm3; Dmax = maximum dose; Dmean = mean dose.

*Note:* Dose parameters are reported in Gy equivalent doses in 2 Gy fractions with an α/β ratio of 3.

**Table 4:** Spearman’s correlation of dosimetric parameters and problematic receptive anal intercourse after prostate radiation therapy.

Dosimetric parameter*	Orgasm ability, ρ	Orgasm pleasure, ρ	Anal pain, ρ
Anal canal			
Dmax (D0.03cm3)	−0.63†	−0.56†	−0.01
Dmean	−0.57†	−0.50†	−0.04
D2cm3	−0.63†	−0.51†	0.09
Levator ani			
Dmax (D0.03cm3)	−0.06	−0.16	0.17
Dmean	−0.58†	−0.48†	−0.02
D2cm3	−0.57†	−0.57†	−0.01
Erectile tissues			
Dmax (D0.03cm3)	−0.61†	−0.41	
Dmean	−0.68†	−0.46	
D2%	−0.61†	−0.39	
Internal pudendal arteries			
Dmax (D0.03cm3)	−0.52‡	−0.55†	
Dmean	−0.29	−0.16	
D2%	−0.41	−0.43	
Neurovascular bundles			
Dmax (D0.03cm3)	0.05	0.05	
Dmean	0.58	0.58	
D2%	−0.23	−0.33	

*Abbreviations:* D0.03cm3 = minimum dose to the hottest 0.03 cm3; D2% = minimum dose to the hottest 2%; D2cm3 = minimum dose to the hottest 2 cm3; Dmax = maximum dose; Dmean = mean dose; ρ = Spearman’s correlation coefficient.

⁎ The relationship between dosimetric parameters and receptive anal intercourse outcome T-scores was assessed with Spearman's correlation. Strong correlations: |ρ| ≥ 0.6 (*p* < .1); moderate correlations: 0.59 ≥ |ρ| ≥ 0.4 (*p* < .1).

† *p* < .05.

‡ *p* < .1.

In exploratory logistic regression analyses of dichotomized outcomes, higher doses to the anal canal, levator ani, IPAs, and erectile tissues were associated with higher odds of dysfunctional orgasm ability. Dose thresholds (Gy EQD2[3]) corresponding to an estimated 10% probability of dysfunction are outlined in Table 5.

**Table 5:** Predicted risk of problematic receptive anal intercourse after prostate radiation therapy by dosimetric parameter.

	Dysfunctional orgasm ability		Dysfunctional orgasm pleasure
Dosimetric parameter	Odds ratio (90% CI)†	10% risk, Gy	Odds ratio (90% CI)†
Anal canal			
Dmax (D0.03cm3)	1.08 (0.99, 1.18)*	40	1.00 (0.95, 1.06)
Dmean	2.19 (1.07, 4.50)*	9	1.25 (0.91, 1.73)
D2cm3	1.18 (1.02, 1.38)*	4	1.06 (0.97, 1.17)
Levator ani			
Dmean	1.66 (1.01, 2.73)*	31	1.05 (0.94, 1.17)
D2cm3	1.07 (0.99, 1.17)		1.02 (0.98, 1.07)
Erectile tissue			
Dmax (D0.03cm3)	1.05 (1.00, 1.10)*	20	
Dmean	1.16 (1.01, 1.32)*	5	
D2%	1.04 (1.00, 1.08)		
Internal pudendal arteries			
Dmax (D0.03cm3)	1.09 (1.01, 1.18)*	35	1.03 (0.99, 1.08)

*Abbreviations:* D0.03cm3 = minimum dose to the hottest 0.03 cm3; D2% = minimum dose to the hottest 2%; D2cm3 = minimum dose to the hottest 2cm3; Dmax = maximum dose; Dmean = mean dose.

⁎ *p* < .1.

† The relationship between dosimetric parameters and dichotomized outcomes was assessed using logistic regression. Outcomes were dichotomized to dysfunction/function based on a clinically meaningful difference of |T-score| ≥ 3. T-scores ≤ 47 indicated dysfunctional orgasm ability/pleasure.

## Discussion

This exploratory study provides the first evidence that radiation dose to RAI functional anatomy is associated with problematic RAI outcomes. These findings support an anatomic basis for both pleasurable and problematic RAI,^6^ expanding the current oncosexual framework beyond penile erectile function.^5^, ^6^, ^7^

Despite not being considered a distinct organ at risk during prostate cancer radiation treatment planning, the anal canal was strongly associated with both orgasm ability and pleasure, highlighting its role as an erotogenic sexual organ.^6^ Notably, an anal canal Dmean of approximately 9 Gy was associated with a 10% risk of orgasm ability dysfunction, substantially lower than the <40 Gy threshold recommended to mitigate fecal incontinence.^24^ These findings suggest that anal canal dose constraints derived from defecation function endpoints may be insufficient surrogates for problematic RAI outcomes.^5^^,^^6^

Observed dose-outcome associations are consistent with anal canal functioning as both a serial and parallel organ.^17^ Focal damage leading to anal canal stenosis may prevent RAI^6^ altogether, consistent with serial organ behavior, whereas diffuse injury to the anal canal and sphincters may reduce contractile function and capacity for pleasurable receptive stimulation,^6^ consistent with parallel organ behavior. Recognition of this dual-response pattern underscores the importance of incorporating RAI outcomes to better characterize radiation-related problematic RAI and inform more personalized radiation treatment planning.

In addition to the anal canal, problematic RAI was associated with dose to the sexual organs at risk already considered in prostate radiation therapy, including the IPAs. We observed a 10% risk of problematic RAI at an IPA Dmax of 35 Gy at 2.3 years posttreatment, consistent with prior data demonstrating a 13% risk of erectile dysfunction at 2 years when IPA D2% exceeds 36 Gy.^2^^,^^4^ These findings support a shared vascular mechanism, in which off-target radiation to the IPAs and their branches may compromise genitopelvic perfusion and engorgement required for orgasm.^6^

Emerging technologies, including heavy-particle therapy,^25^ adaptive image guided radiation therapy,^2^^,^^3^^,^^11^ and dose-reduction medical devices such as perirectal spacers,^26^ have improved dose precision and reduced toxicity, including erectile dysfunction.^2^^,^^3^^,^^11^^,^^25^^,^^26^ Given the importance of the anal canal alongside established sexual structures in RAI,^6^ inclusion of RAI endpoints is needed to determine the utility of these strategies in mitigating problematic RAI.

Our results should be interpreted as preliminary. The small sample size limited the assessment of potential confounders and effect modifiers and likely resulted in overparameterized logistic regression models, with odds ratio estimates that should be interpreted cautiously. Additionally, the small sample size may have limited our ability to detect some associations. For example, the lack of association between neurovascular bundle dose and RAI outcomes, while likely multifactorial (eg, close proximity of the neurovascular bundles to the prostate), may reflect the small sample size. The use of an exploratory threshold (*p* value < .1) further increases the risk of false positives.

Despite uniform assessment of RAI, the cohort included only gay and bisexual cisgender men, limiting generalizability to other populations with a prostate who engage in RAI, such as gender minorities (eg, transfeminine individuals) and heterosexual cisgender men.^5^^,^^6^^,^^27^ The cohort was also predominantly non-Hispanic White, further limiting generalizability and our ability to explore whether psychosocial and contextual factors that may vary by race and ethnicity, such as minority stress, health care access, and cultural attitudes toward RAI, may influence RAI after prostate radiation therapy.^6^

Because this analysis evaluated posttreatment dose-outcome associations without baseline RAI function data, within-person change in RAI function could not be assessed, and the observed associations should be interpreted as hypothesis-generating rather than causal. Preferred sexual behaviors may also change in response to treatment itself, including shifts between receptive and insertive anal intercourse, further complicating the temporal interpretation of RAI function. Individuals who discontinued RAI after treatment may be underrepresented, which could introduce selection bias. Assessments obtained ≥6 months after radiation therapy may capture a mixture of ongoing radiobiologic injury and recovery, limiting inference of longitudinal change and the persistent effects of radiation on RAI.

Radiation therapy delivery was heterogeneous, with distinct modality-specific dose distributions, fractionation schemes, and delivery mechanics. Observed associations may therefore reflect mixed modality-specific injury mechanisms rather than radiation dose alone. Brachytherapy involves intraprostatic implantation and produces steep intraprostatic dose gradients, which may differentially affect prostate-mediated aspects of sexual function compared with external beam radiation therapy. While brachytherapy may be associated with lower rates of anodyspareunia due to relative sparing of the anal canal, it may be associated with orgasm-associated pain or dysfunction during RAI (dysorgasmia).

Larger longitudinal studies with longer follow-up in individuals with prostate cancer and other pelvic malignancies (eg, anorectal cancers^6^) are needed to clarify the dose-response relationship underlying radiation-related problematic RAI.

## Conclusions

This study provides the first evidence on the association between radiation dose to RAI functional anatomy and RAI outcomes, highlighting an anatomic basis for pleasurable RAI and offering novel insights into the pathophysiology of problematic RAI. After discussing sexual behaviors, including RAI, prostate radiation oncologists may consider incorporating the study findings into counseling, shared decision-making, and treatment planning to help mitigate problematic RAI and improve quality of life for individuals who engage in RAI.^7^^,^^12^^,^^28^ While exploratory, this study helps establish an anatomically based oncosexual framework for more inclusive and equitable prostate cancer care.

## Disclosures

Daniel R. Dickstein reports financial support was provided by Conquer Cancer Foundation (ASCO). Deborah C. Marshall reports a relationship with National Institutes of Health that includes: funding grants. Andrew Jackson reports a relationship with National Institutes of Health that includes: funding grants. Kathryn E. Flynn reports a relationship with National Institutes of Health that includes: funding grants; reports a relationship with University of Wisconsin that includes: consulting; reports a relationship with Mount Sinai that includes: consulting; reports a relationship with Novartis that includes: advisory board participation and consulting; and reports a relationship with Inhibikase that includes: consulting. The other authors declare that they have no known competing financial interests or personal relationships that could have appeared to influence the work reported in this paper.

## Appendix Supplementary materials

Supplementary materials Supplementary Materials Supplementary material associated with this article can be found in the online version at xxx. Image, application 1
