# Supplementary material for: Dosimetric Predictors of Problematic Receptive Anal Intercourse After Prostate Radiation Therapy
Source: Adv Radiat Oncol. 2026 Apr 3;11(6):102038. doi: 10.1016/j.adro.2026.102038 (PMC13187525; doi:10.1016/j.adro.2026.102038)
Supplement: Supplementary materials — Supplementary material associated with this article can be found in the online version at xxx. [file mmc1.pdf]

Table E1. Patient-Reported Outcomes Measurement Information System (PROMIS) Sexual Function and Satisfaction (SexFS) Version 2.0 Select Item Response Distributions Among Individuals Engaging in Receptive Anal Intercourse After Prostate Radiotherapy

In the past 30 days, how often have you been able to have an orgasm/climax when you wanted?					
Have not tried to have an orgasm/climax in the past 30 days	Never	Rarely	Sometimes	Often	Always
0 (0%)	1 (7%)	2 (14%)	3 (21%)	2 (14%)	6 (43%)
**In the past 30 days, how satisfying have your orgasms or climaxes been?**					
Have not tried to have an orgasm/climax in the past 30 days	Not at all	A little bit	Somewhat	Quite a bit	Very
0 (0%)	1 (7%)	3 (21%)	5 (36%)	1 (7%)	4 (29%)
**In the past 30 days, how much pleasure have your orgasms or climaxes given you?**					
Have not tried to have an orgasm/climax in the past 30 days	Not at all	A little bit	Somewhat	Quite a bit	Very
0 (0%)	1 (7%)	4 (29%)	3 (21%)	2 (14%)	4 (29%)

In the past 30 days, when you have had sexual activity, how much pain have you had in or around your anus or rectum?				
None	A little bit	Some	Quite a bit	A lot
12 (86%)	1 (7%)	1 (7%)	0 (0%)	0 (0%)
**In the past 30 days, when you have had sexual activity, how much discomfort have you had in or around your anus or rectum?**				
None	A little bit	Some	Quite a bit	A lot
10 (71%)	3 (21%)	1 (7%)	0 (0%)	0 (0%)
